# Effects of quadratus lumborum block on perioperative multimodal analgesia and postoperative outcomes in patients undergoing radical prostatectomy

**DOI:** 10.1186/s12871-022-01755-w

**Published:** 2022-07-11

**Authors:** Zhen Hu, Yingjie Zhou, Guohao Zhao, Xinxin Zhang, Chunchun Liu, Huan Xing, Ji Liu, Fen Wang

**Affiliations:** 1 Department of Anesthesiology, Shanghai Tenth People’s Hospital, Tongi University School of Medicine, Shanghai, 200072 People’s Republic of China; 2 Department of Urology, Shanghai Tenth People’s Hospital, Tongji University School of Medicine, Shanghai, Shanghai, 200072 People’s Republic of China

**Keywords:** Comfort, Multimodal analgesia, Postoperative outcome, Quadratus lumborum block, Radical prostatectomy

## Abstract

**Background:**

This study aimed to investigate the effects of ultrasound-guided quadratus lumborum block (QLB) on perioperative multimodal analgesia and postoperative outcomes in patients undergoing radical prostatectomy.

**Methods:**

A total of 80 patients undergoing radical prostatectomy were randomly divided into two groups: general anaesthesia with QLB (QLB group; *n* = 40) and general anaesthesia with sham QLB (normal saline [NS] group; n = 40). QLB or sham QLB was performed before the induction of anaesthesia. Sufentanil was intravenously administered for postoperative analgesia. The primary outcome was the pain score (measured using a numerical rating scale [NRS]) at different time points within 48 h postoperatively. Secondary outcomes included the cumulative dose of sufentanil within 48 h postoperatively, subjective comfort, grip strength, first time of exhaustion, first fluid intake time, time to get out of bed, length of postoperative hospital stay and overall satisfaction. The SPSS software, version 17.0, was used for all statistical analyses.

**Results:**

Postoperative NRS at rest was significantly lower at 2 h (1.7 ± 1.1 versus 3.0 ± 2.1), 4 h (1.8 ± 1.2 versus 4.1 ± 2.3), 6 h (1.9 ± 2 versus 4.4 ± 2) and 12 h (3.5 ± 2.3 versus 5 ± 3.3) and was also lower when coughing at 2 h (2.3 ± 1.1 versus 4 ± 2.1), 4 h (2.3 ± 1. 1 versus 4.3 ± 2) and 6 h (2.4 ± 1.1 versus 5.9 ± 2.3) in the QLB than that in the NS group. The cumulative dose of sufentanil was significantly lower in the QLB than that in the NS group at 4 h, 6 h, 12 h, 24 h and 48 h. The nausea score was significantly lower in the QLB group at 24 h postoperatively, and the first time of exhaustion and time to get out of bed were significantly shorter (*P* < 0.05). The overall satisfaction score was significantly higher in the QLB than in the NS group (4 ± 0.7 versus 2.6 ± 0.9).

**Conclusion:**

Ultrasound-guided bilateral QLB can provide effective postoperative analgesia for patients undergoing radical prostatectomy, reduce the need for sufentanil, facilitate comfort and improve postoperative outcomes. QLB can be a good component of multimodal analgesia.

**Trial registration:**

The clinical trial is registered in the Chinese Clinical Trial Registry (ChiCTR). Current Controlled Trials:ChiCTR1900022009. the date of registration:2019/03/20.

## Background

Open radical prostatectomy is a time-consuming procedure, which causes major trauma and requires the insertion of many indwelling catheters postoperatively. However, patient expectations of minimal postoperative pain and rapid recovery remain unmet. Continuous epidural block and abdominal transverse block are the techniques generally used to achieve analgesia in patients undergoing open radical prostatectomy. However, both methods have their own disadvantages. Epidural block is associated with a high risk of postoperative hypotension and results in transient lower limb weakness postoperatively. Abdominal transverse plane block has unreliable analgesic effects, only provides short-term analgesia. Therefore, they aren’t the best choice as one of the elements of multimodal analgesia. In 2007, Blanco proposed an alternative abdominal wall block procedure-the quadratus lumborum block (QLB) [1]. QLB has been successfully performed to provide postoperative analgesia following caesarean section [2–4], radical colectomy [5], appendectomy, hip joint surgery [6] and paediatric surgery [7, 8] among others. Horosz [9] found that aplication of bilateral bilateral posterior quadratus lumborum block did not reduce opioid consumption after minimally invasive prostatectomy. However, no studies have reported its use as perioperative analgesia for patients undergoing open radical prostatectomy.

We hypothesised that QLB would effectively reduce postoperative pain in patients undergoing open radical prostatectomy, decrease the required sufentanil dose and thereby promote postoperative recovery. Therefore, this study aimed to evaluate the efficacy of ultrasound-guided QLB to provide perioperative analgesia and outcome effects in patients undergoing radical prostatectomy.

## Methods

A total of 80 patients scheduled for radical prostatectomy from March 25, 2019 to April 1, 2020 were recruited for this study. Patients with the following conditions were excluded: 1) ropivacaine allergy, 2) psychiatric illness, 3) skin infection at the proposed puncture site, 4) peripheral neuropathy, 5) history of opioid abuse, 6) chronic pain or 7) inability to complete the pain digital rating scorer inability score pain on the numerical rating scale (NRS). Withdrawal criteria were death within 48 h postoperatively and major intra- or postoperative bleeding.

This clinical trial was started after obtaining approval from the ethics committee of Shanghai Tenth People’s Hospital. Informed consent was obtained from all patients (or family members).

SPSS 17.0 (SPSS Inc., Chicago, IL, USA) was used for random grouping to conceal the random process using envelopes to seal the random numbers, which were strictly assigned based on the order of patient selection. Patients were randomly divided into QLB and sham block groups in a 1:1 ratio. Randomisation was performed inside the operating room. A sealed envelope containing the randomisation code was opened just before administering anaesthesia. Enrolled patients were randomly assigned to receive either general anaesthesia plus QLB with ropivacaine (QLB group, *n* = 40) or plus sham QLB, that is, normal saline (NS group, *n* = 40). A caregiver who was unaware of the experimental protocol prepared the ropivacaine and saline solutions. Subsequently, a preloaded syringe containing 20 ml of the colourless solution was handed over to the anaesthetist. Patients, anaesthesiologists, surgeons and follow-up personnel were blinded of the drugs used.

Systolic blood pressure (SBP), diastolic blood pressure (DBP), mean arterial pressure (MAP), heart rate (HR), electrocardiogram (ECG), bispectral index (BIS) and the degree of peripheral capillary oxygen saturation (SpO_2_) were routinely monitored after the patients entered the operating room. The central venous access was established under local anaesthesia. General anaesthesia was induced with 1.5–2.0 mg/kg of propofol (production batch number: X17052B, AstraZeneca Pharmaceutical Co., Ltd.), 0.4 μg/kg of sufentanil (production batch number: 1180414, Yichang Renfu Pharmaceutical Co., Ltd.) and 0.15 mg/kg of cisatracurium (production batch number: 180702AJ, Jiangsu Hengrui Pharmaceutical Co., Ltd.) via tracheal intubation. Intraoperative BIS was maintained at 40–60.

Anaesthesia was maintained with 4–6 mg/kg/h of intravenous propofol (production batch number: NX190, AstraZeneca Pharmaceutical Co., Ltd.), 0.05–0.2 μg /kg/min of remifentanil (production batch number: 80A05081, Yichang Renfu Pharmaceutical Co., Ltd.) and 0.7–1.5 minimum alveolar concentration (MAC) of inhaled sevoflurane (production batch number: 18070531, Shanghai Hengrui Pharmaceutical Co., Ltd.). Cisatracurium at a dose of 4–6 mg/h was administered through intermittent intravenous injection. Remifentanil and sevoflurane concentrations were adjusted based on varied vital signs. For analgesia, all patients were intravenously administered 40 mg of parecoxib (production batch number: W27811, Pfizer Pharmaceutical Co., Ltd.) and 12.5 mg of dolasetron mesylate (production batch number: 18062171, Liaoning Haisi Pharmaceutical Co., Ltd.). The intravenous and inhaled anaesthetics were discontinued when the subcutaneous tissue had been sutured. The endotracheal tube was removed after spontaneous breathing was resumed and consciousness level was returned.

Patients with high SBP (≥140 mmHg) for the first 2–3 min intraoperatively were administered sufentanil at a dose of 5-10 μg. However, if SBP remained high, 0.3–0.5 mg of perdipine was intravenously infused and readministered as necessary. Patients with low SBP (< 90 mmHg) for the first 2–3 min intraoperatively were intravenously administered 40-80 μg of phenylephrine. If there was no response, the same medications were readministered, and 200 ml of colloidal solution was added and rapidly infused. If HR was higher than 100 beats/min, 1 mg/kg of esmolol was intravenously administered and repeated as necessary, whereas if HR was lower than 45 beats/min, 0.5 mg of atropine was intravenously administered and repeated as necessary.

Postoperatively, 150 μg of sufentanil dissolved in 100 ml NS was intravenously infused at 2 ml/h for analgesia in both groups, and patient-controlled analgesia was achieved with a dose of 0.5 ml and a locking interval of 15 min. Patients with severe postoperative pain (NRS ≥ 7) were administered 50 mg of meperidine intramuscularly.

**Fig. 1:**
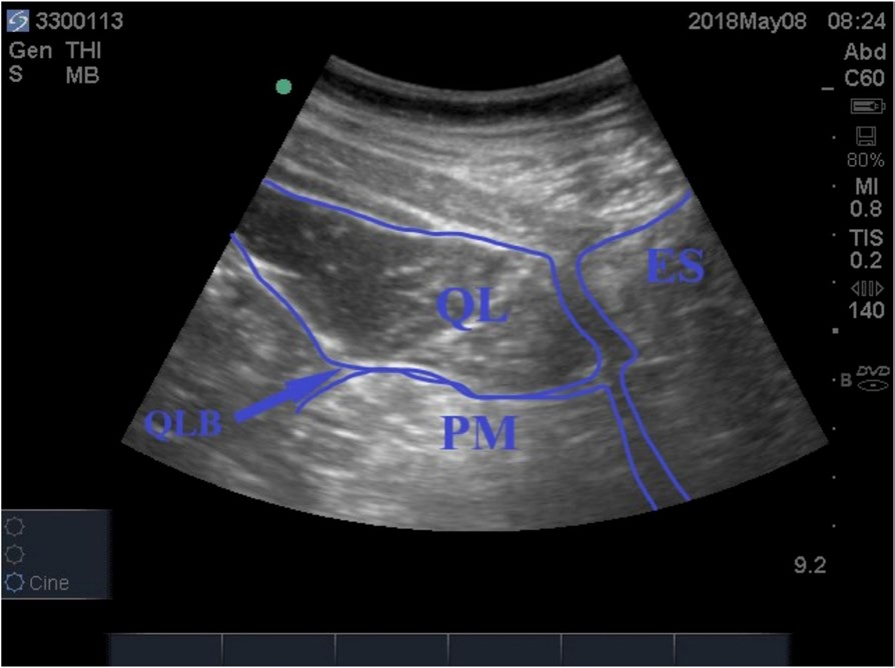
Diagram of ultrasound-guided quadratus lumborum block. *Note*: *QL* Quadratus lumbar muscle, *QLB* quadratus lumborum block, *PM* psoas muscle, *ES* erector spinae. The arrow tip indicates the drug injection point

The QLB group underwent bilateral QLB before the induction of general anaesthesia. The QLB procedure has been previously described [10]. Briefly, patients were placed in the right lateral position and routinely disinfected and draped. A portable, coloured, two-dimensional ultrasound machine (Bothell, FUJIFILM SonoSite, USA) equipped with a low frequency (5–2 MHz) convex array probe was used for guidance. First, the probe was placed horizontally above the anterior part of the iliac crest, and the external oblique, internal oblique and transverse abdominis muscles were identified. Subsequently, the probe was moved to the back until the quadratus lumbar muscle, psoas muscle, erector spinae and transverse processes of the vertebrae were visible. A 22-G, 8-cm-long nerve plexus stimulation needle (Béron, USA) was inserted from the dorsal to the ventral side. The needle was advanced until the tip was located to the fascia between the quadratus lumborum and psoas muscles (as indicated by the blue arrow in Fig. 1). Correct needle tip positioning was confirmed by injecting 1 ml of NS. Subsequently, a 20 ml mixture comprising 75 mg of ropivacaine (production batch number: 180611CA, Jiangsu Hengrui Pharmaceutical Co., Ltd.) and 25 μg of dexmedetomidine (production batch number: 181012BP, Jiangsu Hengrui Pharmaceutical Co., Ltd.) was injected [11]. The patients were then shifted to the left lateral position, and the procedure was repeated for the left QLB. The same procedure was administered to patients in the NS group; however, 20 ml of 0.9% NS was injected instead of the anaesthetic mixture. All operations were performed by the same group of experienced urologists. All QLBs were performed by the same experienced anaesthesiologist. The average operative time was 12.5 minutes.

**Fig. 2:**
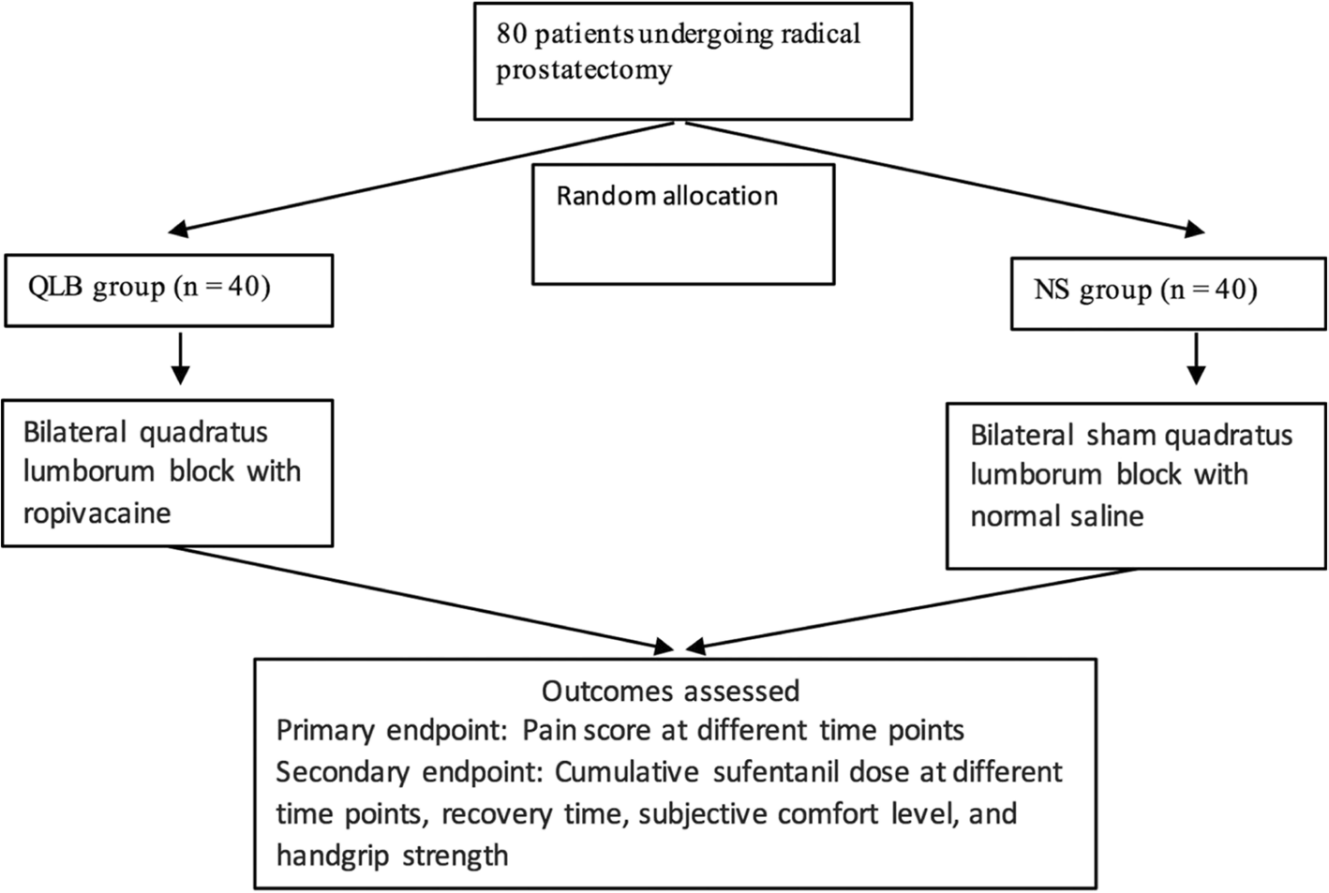
Trials flow chart. Eighty patients who underwent open radical prostatectomy were randomly divided into QLB group and NS group, 40 cases in each group. The QLB group underwent bilateral quadratus lumborum block before induction of general anesthesia, and the NS group was injected with equal volume of normal saline. NRS, cumulative dosage of sufentanil and clinical outcomes were evaluated at different time points after operation. *Note***:** *QLB group* Quadratus lumborum block group, *NS group* normal saline group, *NRS* numerical rating scale

The primary end-point was the NRS score at different time points during the first 48 h postoperatively. NRS scores at rest and when coughing were recorded at 2, 4, 6, 12, 24 and 48 h postoperatively. Secondary end-points included the intraoperative remifentanil and sufentanil doses, subjective comfort, handgrip strength and recovery parameters (first time of exhaustion, first fluid intake time, time to get out of bed and length of postoperative hospital stay). Cumulative doses of sufentanil at the same time points were recorded. Moreover, 50 mg of meperidine was considered equivalent to 5 μg of sufentanil. The degrees of anxiety, thirst, hunger, nausea and fatigue were each scored on a scale of 0 to 10, (0, no discomfort; 1–3, mild discomfort; 4–6, severe discomfort; 7–9, severe discomfort and 10, unbearable discomfort). The total score was used to measure subjective comfort. Handgrip strength was standardised by expressing it as a function of body weight using the following formula: *(grip strength [kg] / body weight [kg]) × 100*. Handgrip strength was tested on the same upper limb. Subjective comfort, handgrip strength were evaluated preoperatively and at 24, 72 and 168 h postoperatively. The first time of exhaustion (time from the end of the operation to the first exhaust), first fluid intake time (time from the end of the operation to the first fluid intake), time to get out of bed (time from the end of the operation to the first off-bed activity) and postoperative hospital stay (days from the end of the operation to discharge) were recorded. Overall satisfaction was evaluated at 48 h postoperatively in both groups and graded as follows: 5, very satisfied; 4, satisfied; 3, somewhat satisfied; 2, dissatisfied and 1, very dissatisfied. The primary and secondary outcomes were assessed and recorded face-to-face at 2, 4, 6, 12, 24, 48, 72, and 168 hours postoperatively by nursing staff who were unaware of the grouping status. The trial flow chart was shown in Fig. 2.

Statistical analysis: The SPSS software, version 17.0, was used for all statistical analyses. Normally distributed continuous data were expressed by means (standard deviation) and comparisons were made between groups using the independent samples *t-*test. Continuous data of repeated measurements in the same group were compared using repeated-measures analysis of variance (ANOVA). Multivariate ANOVA was used to compare between groups at the same time points. Count data were expressed as percentages and compared using the chi-square test. *P* ≤ 0.05 was considered statistically significant.

## Results

A total of 80 patients were enrolled and randomised into two groups (*n* = 40). The age and BMI of patients ranged from 51 to 83 years and from 18.5 to 31.7 kg/m^2^, respectively. Demographic characteristics, pathological type post-operation, fluid volume intraoperation, blood loss, operation time and the time in the postoperative anesthesia care unit (PACU) were not significantly different between the two groups (*P* > 0.05; Table 1).

**Table 1:** Demographic characteristics and surgical parameters of the two groups (*n* = 40, $\overline{x}\pm s$)

	QLB group	NS group	*P* value
Age (y, $\overline{\ x}\pm s$)	68 ± 9	70 ± 7	0.47
BMI (kg/m^2^, $\overline{x}\pm s$)	24 ± 5	24 ± 3	0.25
ASA (n, I/II/III)	3/30/7	5/29/6	0.45
Pathology(n, I/II/III/IV)	26/12/2/0	24/13/3/0	0.79
Bleeding (ml, $\overline{x}\pm s$)	189 ± 53	192 ± 47	0.45
Fluid volume (ml, $\overline{\ x}\pm s$)	1750 ± 104	1880 ± 88	0.60
Operative time (min, $\overline{x}\pm s$)	175 ± 36	172 ± 34	0.36
PACU time (min, $\overline{x}\pm s$)	63 ± 12	60 ± 9	0.55

*Note*: *QLB group* Quadratus lumborum block group, *NS group* normal saline group. *BMI* body mass index, *ASA* American Society of Anesthesiologists, *PACU* postoperative anesthesia care unit

**Fig. 3:**
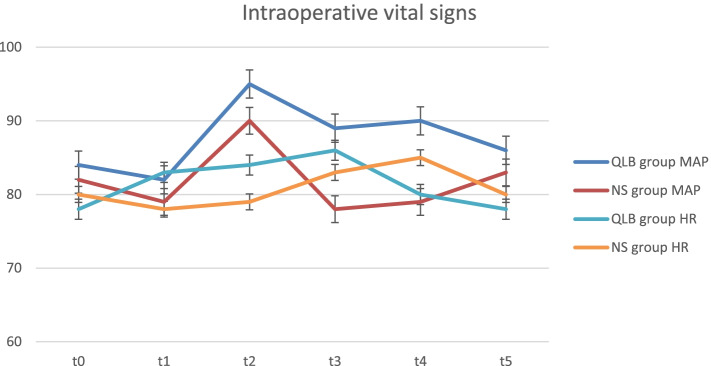
Intraoperative vital signs**.** *Note***:** *QLB group* Quadratus lumborum block group, *NS group* normal saline group, *MAP* mean arterial pressure, *HR* heart rate, *t*_*0*_ 10 minutes after entering the operating room, *t*_*1*_ surgery begins, *t*_*2*_ 5 minutes after surgery, *t*_*3*_ 30 minutes after surgery, *t*_*4*_ 60 minutes after surgery, *t*_*5*_ at the end of surgery

There were no significant differences in mean arterial pressure and heart rate at baseline, at the start of surgery, at 5 minutes after surgery, at 30 minutes after surgery, at 60 minutes after surgery, and at the end of surgery between the two groups (*P* > 0.05; Fig. 3).

**Fig. 4:**
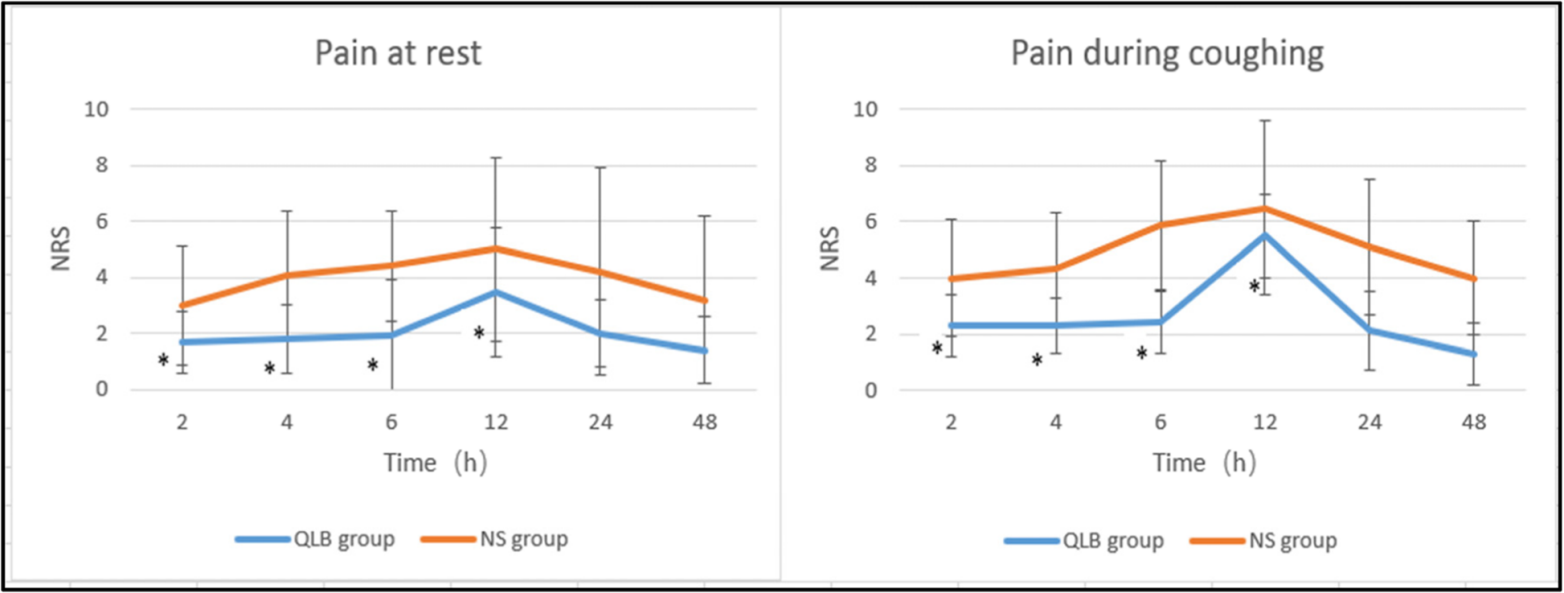
**A** Comparison of pain at rest between the two groups. **B** Comparison of pain when coughing between the two groups. *Note QLB group* Quadratus lumborum block group, *NS group* normal saline group, *NRS* numeric rating scale. *P<0.05

NRS scores at rest and when coughing were significantly lower in the QLB than in the NS group at 2, 4, 6 and 12 h postoperatively (*P* < 0.05 for all; Fig. 4A and B).

No statistical difference in the amount of remifentanil was observed between the two groups (*P* = 0.57). The cumulative dose of sufentanil was significantly lower in the QLB than that in the NS group at 4, 6, 12, 24 and 48 h postoperatively (*P* < 0.05, Table 2).

**Table 2:** Cumulative dosage of remifentanil and sufentanil (μg, *n* = 40)

Group	remifentanil	sufentanil (T_1_)	sufentanil(T_2_)	sufentanil (T_3_)	sufentanil (T_4_)	sufentanil (T_5_)	sufentanil (T_6_)
QLB group	950 ± 120	6.1 ± 0.5	13.9 ± 3.4*	22.5 ± 5.7*	40 ± 10*	78 ± 71.7*	151 ± 9*
NS group	1044 ± 217	7.1 ± 0.6	16.4 ± 6.9	29.0 ± 6	48.3 ± 10	86.5 ± 24	160 ± 10
*P* value	0.57	0.34	0.04	0.03	0.01	0.04	0.03

*Note*: *QLB group* Quadratus lumborum block group, *NS group* normal saline group, ^*^P < 0.05. T_1_: 2 h, T_2_: 4 h, T_3_: 6 h, T_4_: 12 h, T_5_: 24 h and T_6_: 48 h postoperatively

**Fig. 5:**
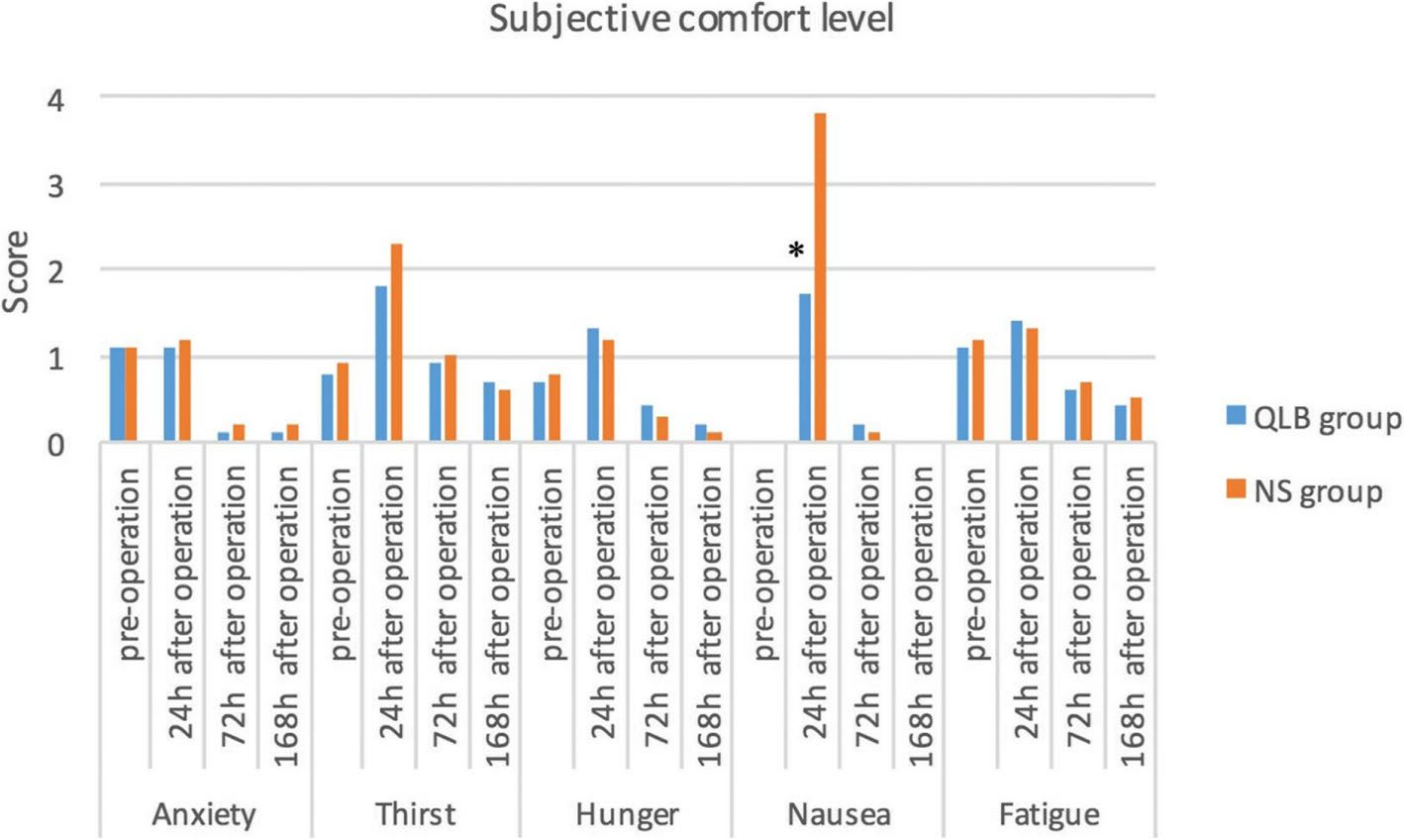
Comparison of scores for different elements of comfort in the two groups. *Note: QLB group* Quadratus lumborum block group, *NS group* saline group. *P<0.05

The nausea score was significantly lower in the QLB than that in the NS group at 24 h postoperatively (*P* < 0.05, Fig. 5). Handgrip strength was not significantly different between the two groups preoperatively or at 24, 72 and 168 h postoperatively (51, 46, 48 and 50, respectively, in the QLB group versus 52, 47, 48 and 51, respectively, in the NS group; *P* > 0.05 at all time-points).

The first time of exhaustion and time to get out of bed were significantly shorter in the QLB than in the NS group (*P* < 0.05; Table 3).

**Table 3:** Comparison of recovery parameters between the QLB and NS groups (*n* = 40)

	QLB group	NS group	*P* value
First time of exhaustion(h)	28.2 ± 12.4*	30.4 ± 10.5	0.047
Time to get out of bed (h)	33.7 ± 19.2*	40.9 ± 16.2	0.03
Time to first intake of water (h)	17.4 ± 7.5	15.3 ± 6.8	0.15
Time to first intake of food (h)	41.1 ± 16	38.5 ± 14	0.25
Postoperative hospital stay (day)	12.5 ± 5.7	14.2 ± 6.5	0.45

*Note*: *QLB group* Quadratus lumborum block group, *NS group* normal saline group, ^*^P < 0.05

The overall satisfaction score at 48 h postoperatively was significantly higher in the QLB than in the NS group (4 ± 0.7 versus 2.6 ± 0.9, *P* < 0.05).

## Discussion

Ultrasound-guided QLB can be performed using three methods. First, in the QLB1 method, the drug is injected at the anterior aspect of the quadratus lumbar muscle, the junction of the quadratus lumbar muscle and the transversalis fascia. Second, in the QLB2 method, the drug is injected posterior to the quadratus lumbar muscle, between the quadratus lumbar muscle and erector spinae. Third, in the QLB3 method, the drug is injected in front of the quadratus lumbar muscle, between the quadratus lumbar and psoas muscles and deep into the anterior layer of the thoracolumbar fascia. In a cadaveric study, Carline et al. [12–14] investigated the diffusion of dyes injected using these three methods and found that dyes injected using QLB1 and QLB2 methods were mainly diffused to the nerves in the transversalis fascia and sometimes to the skin of the anterior abdomen or deep into the back muscles. In QLB3, the dye diffused to the L_1_-L_3_ nerve root distribution area and spread to the psoas and lumbar muscles. Therefore, QLB3 was concluded to provide the most effective block and used in this study.

Previous studies [15] have demonstrated that local anaesthetics spread to the thoracolumbar paraspinal space and thoracodorsal fascia, and hence, the blocking effect to the quadratus lumbar muscle is better than that of the abdominal transverse plane block with block duration being equivalent to the epidural block [16, 17]. The local anaesthetics recommended for QLB included 0.25% levobupivacaine, 0.125% bupivacaine and 0.25–0.375% ropivacaine (20–30 ml) [18]. In this study, 20 ml of 0.375% ropivacaine was used [11], with 50 μg of dexmedetomidine added to strengthen and prolong the blocking effect [19]. Horosz et al. used 30 ml of 0.375% ropivacaine for quadratus lumborum block, whereas we added 25μg of dexmedetomidine as an adjuvant to 20 ml of 0.375% ropivacaine. Studies [20, 21] have shown that dexmedetomidine is a potential local anesthetics adjuvant that can exhibit a facilitatory effect when administered intrathecally as part of spinal anaesthesia or peripherally as part of a brachial plexus block. Sensory block duration and motor block duration and time to first analgesic request were prolonged for both intrathecal and brachial plexus block. This may be one of the factors that led to the difference between the results of our study and Horosz et al.

In this study, NRS at rest were significantly lower in the QLB than in the NS group at 4 and 6 h postoperatively, and NRS when coughing was significantly lower in the QLB group at 2, 4, 6 and 12 h postoperatively. The required sufentanil dosage was significantly lower in patients administered with QLB than in those administered with NS at 4, 6 and 12 h postoperatively. The duration of sensory nerve block with ropivacaine is within 10–12 h, and the addition of dexmedetomidine can extend the blocking duration to 15 h [19]. In this study, the QLB group exhibited much better pain control during movement than that exhibited by the NS group at 12 h postoperatively. When the effects of ropivacaine subsided, the difference in pain scores between the two groups gradually diminished. Because of the analgesic effect of ropivacaine, the need for sufentanil was much lower in patients with QLB [22], which consequently reduced the occurrence of postoperative nausea. Better pain relief also resulted in earlier getting out of bed and passage of flatus.

Handgrip strength is highly correlated with the overall body strength and indirectly reflects a person’s general health. In this study, no significant difference was observed in handgrip strength between the QLB and NS groups, indicating that lumbar muscle block does not adversely affect the overall muscle strength.

In April 2018, Myles and other scholars proposed a standardised method for evaluating perioperative outcomes, with emphasis on patient experience and comfort, and identified six end-points: postoperative pain intensity (at rest and during movement) during the first 24 h, nausea and vomiting (0–6 h, 6–24 h and total duration), two ‘quality-of-recovery’ scores (quality-of-recovery [QoR] scales [23], QoR score or QoR-15 [24]), time to gastrointestinal recovery, time to an out-of-bed activity and sleep quality [25]. Accordingly, this study assessed pain intensity during rest and movement, nausea and vomiting in the first 6–24 h postoperatively, time to gastrointestinal recovery (time to the first flatus and oral intake) and the quality of recovery (time to independent out-of-bed activity) as outcome indicators.

This quality study has some limitations. A major limitation was that the efficacy of QLB was not compared with that of abdominal transverse fascia and epidural blocks. Furthermore, this is a single-centre study, and its results cannot be generalized. Because this study is a preliminary exploratory study, and the sample size is not the main consideration, the conclusions of the study are only preliminary. In the future, we need to increase the sample size to further verify the potential benefit of QLB on the outcomes of radical prostatectomy patients.

## Conclusions

Ultrasound-guided bilateral QLB can provide effective postoperative analgesia to patients undergoing radical prostatectomy, reduce the need for sufentanil, improve comfort and promote outcomes. QLB can be a good component of multimodal analgesia.

## Data Availability

All data and materials obtained in this research are true and effective.

## Abbreviations

QLB: quadratus lumborum block
QLB group: quadratus lumborum block group
NS: normal saline
NS group: normal saline group
NRS: numerical rating scale
SBP: Systolic blood pressure
DBP: diastolic blood pressure
MAP: mean arterial pressure
HR: heart rate
ECG: electrocardiogram
BIS: bispectral index
SpO_2_: peripheral blood oxygen saturation
MAC: minimum alveolar concentration
BMI: body mass index
ASA: American Society of Anesthesiologists
PACU: postoperative anesthesia care unit
